# The association between HDL‐C and stroke in the middle‐aged and elderly: A cross‐sectional study

**DOI:** 10.1002/brb3.2901

**Published:** 2023-02-07

**Authors:** Yang Hu, Min Song, Dongmei Wu, Yuqing Zhang, Gongbo Li, Haiyan Luo

**Affiliations:** ^1^ Department of Neurology The Second Affiliated Hospital of Chongqing Medical University Chongqing China

**Keywords:** aged, cholesterol, HDL, middle aged, stroke

## Abstract

**Background:**

Previous epidemiological studies have indicated that high‐density lipoprotein cholesterol (HDL‐C) is inversely associated with the risk of cardiovascular disease. However, this issue has aroused controversy in recent years. Besides, the relationship between HDL‐C and the risk of total stroke in sex and race is less clear. Thus, we aimed to examine the association between different ranges of HDL‐C and the risk of total stroke in adults over 40 years old.

**Methods:**

This cross‐sectional study was conducted on a sample of 8643 participants (4222 men and 4421 women) aged ≥40 years old from the National Health and Nutrition Examination Survey 2007–2016. HDL‐C was an independent variable and stroke was a dependent variable in this study, with the other variables as potential effect modifiers. To examine the associations between them, we used multivariate logistical regression models and smooth curve fittings, as well as subgroup analyses.

**Results:**

HDL‐C was inversely associated with stroke when HDL‐C was less than 1.55 mmol/L (odds ratio [OR] = 0.36, 95% confidence interval [CI] :0.21–0.62, *p* < .05). However, above 1.55 mmol/L, the incidence of stroke was not significant (OR = 1.29, 95% CI: 0.79–2.09, *p*>.05). Stratified by race/ethnicity and sex, the subgroup analyses demonstrated that HDL‐C was inversely associated with stroke in men and Whites, but no significant differences among women, Mexicans, blacks, and other races.

**Conclusion:**

We found a nonlinear relationship between HDL‐C and total stroke. Our study reveals a range of inverse associations between HDL‐C and stroke (HDL‐C<1.55 mmol/L), especially among men and Whites. This finding suggested that maintaining an appropriate HDL‐C range may be beneficial in reducing the incidence of stroke.

## Graphical abstract

(1) After adjusting for age, sex, race/ethnicity, LDL‐C, cholesterol, Triglycerides, HbA1c, income to poverty ratio, education level, smoking status, alcohol consumption, body mass index (BMI), diabetes, hypertension, We found a non‐linear relationship between HDL‐C and stroke. (2) HDL‐C was inversely associated with stroke when HDL‐C was less than 1.55 mmol/L (OR = 0.36, 95% CI :0.21–0.62 *P* < 0.05) after adjusting for other confounders. (3) In subgroup analyses stratified by sex and race/ethnicity, HDL‐C was inversely associated with stroke in men and Whites.

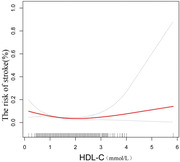

## 1 INTRODUCTION

Stroke is the second leading cause of death and a major contributor to disability worldwide. It affects roughly 13.7 million people and kills around 5.5 million annually (GBD 2016 Stroke Collaborators, 2019). The most common and controllable risk factors for stroke include high blood pressure, diabetes, dyslipidemia, smoking, and obesity (O'donnell et al., 2010). High‐density lipoproteins (HDL) are a type of lipoproteins that carry cholesterol in the blood. Elevated concentration of serum high‐density lipoprotein cholesterol (HDL‐C) protects against cardiovascular disease through a variety of mechanisms, including reverse cholesterol transport, anti‐inflammatory, antioxidant, and antithrombotic effects (Navab et al., 2009; Rosenson et al., 2013). However, studies investigating the association between HDL‐C and stroke remain limited and controversial.

Several previous studies showed that higher HDL‐C was associated with a lower risk of stroke (Reina et al., 2015; Zhang et al., 2012). While other studies showed that the levels of HDL‐C were not associated with stroke (Bowman et al., 2003; Rohatgi et al., 2014). In contrast, a recent meta‐analysis reported that high levels of HDL‐C may be associated with a higher risk of stroke (Wang et al., 2013). Thus, the claim that increasing HDL‐C treatment is beneficial for stroke is being challenged.

To the best of our knowledge, few studies have reported the exact dose‐response relationship between HDL‐C and total stroke with different sex and races. Therefore, we aimed to examine the association between the different HDL‐C levels and total stroke in people older than 40 years old using a large‐scale public database from the National Health and Nutrition Examination Survey (NHANES). Our findings could facilitate the development and promotion of blood lipid prevention strategies aimed at reducing the risk of stroke.

## 2 MATERIALS AND METHODS

### 2.1 Data collection and study population

**FIGURE 1:**
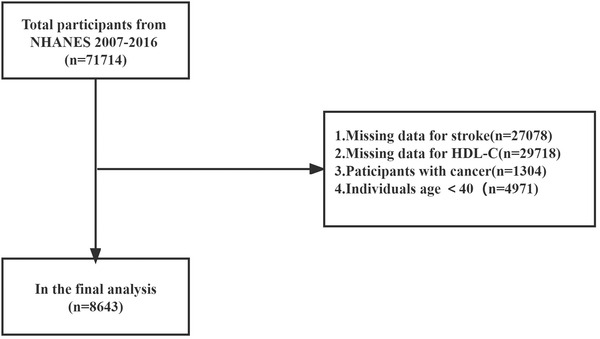
Flow diagram of the screening and enrollment of study participants. Total people from the National Health and Nutrition Examination Survey (NHANES) 2007−2016.

The NHANES is a population‐based national survey that collects health and nutrition data in the United States in biennial cycles. Besides, the NHANES is a cross‐sectional study designed to produce a nationally representative sample of the U.S. population by using a multifaceted probability design. In mobile examination centers, following a standardized home interview, a physical examination and biological specimen collection are conducted. The NHANES data are freely available to researchers throughout the world on the internet. Information on HDL‐C as well as stroke measures in five cycles (2007–2016) were combined and used in this analysis. Generally, a total of 71,714 individuals participated in the NHANES from 2007 to 2016. We defined middle‐aged and elderly adults as aged ≥40 years (Feifel & Strack, 1989). Participants with missing stroke data (*n* = 27078) and HDL‐C data (*n* = 29718), as well as participants with cancer (*n* = 1304), and who were under the age of 40 (*n* = 4971) were excluded. In our analysis, we included 8643 participants (Figure 1). The National Center of Health Statistics Ethics Review Board approved the study, and written informed consent was obtained from each participant.

### 2.2 Definition of stroke

In the NHANES, participants were asked whether and when they had a prior stroke. Participants were asked, “Has a doctor or other health professional ever told you that you had a stroke?” In the United States, self‐reported measures for stroke are fairly accurate among the general population and have been previously used in epidemiological studies with the NHANES data (Lin et al., 2015).

### 2.3 Assessment of HDL‐C

HDL‐C was measured by the direct immunoassay method during 2007–2012. During the 2013–2016 cycle, neither the lab method nor the lab location was changed. However, the HDL‐C lab equipment was changed. In the 2007–2012 cycle, the analyte was measured using the Roche Modular P chemistry analyzer. The analyte was measured using Roche Cobas 6000 and Roche modular P chemistry analyzers from 2013 to 2016.

Some large‐scale studies demonstrated that HDL‐C ≥2.07 mmol/L (≥80 mg/dl) or ≥2.32 mmol/L (≥90 mg/dl) may increase the risk of cardiovascular disease events (Ko et al., 2016; Madsen et al., 2017; Wilkins et al., 2014). According to a Japanese community‐based cohort study, HDL‐C levels between 1.56 and 2.06 mmol/L (60–79 mg/dl) were associated with a decreased risk of coronary heart disease morbidity compared to those with lower levels between 1.04 and 1.55 mmol/L (40−60 mg/dl). In order to examine the association between HDL‐C and stroke, we divided the HDL‐C levels into five categories: HDL‐C <1.04 mmol/l (<40 mg/dl), 1.04–1.56 mmol/l (40–59 mg/dl), 1.56–2.07 mmol/l (60–79 mg/dl), 2.07–2.32 mmol/l (80–89 mg/dl), and ≥2.32 mmol/L (≥90 mg/dl).

### 2.4 Covariates

Demographic variables included age, sex, race/ethnicity, low‐density lipoprotein cholesterol (LDL‐C), cholesterol, triglycerides, glycated haemoglobinA1c (HbA1c), income‐to‐poverty ratio, smoking status, education level, body mass index (BMI), alcohol consumption, diabetes, hypertension, and cancer. Race/ethnicity was categorized into Mexican American, other Hispanic, non‐Hispanic White, non‐Hispanic Black, and other races. Education level was defined as below high school, high school, and above high school. The DxC800 measures serum cholesterol concentrations by using a timed‐endpoint method. LDL‐C was converted to categorical variables considering the impact of missing LDL data on sample size. HbA1c levels were assessed with high‐performance liquid chromatography, standardizing the procedure to the Diabetes Control and Complications assay. The poverty ratio index was used to define poverty. Income‐to‐poverty ratio was set at the following cut‐points: <1.99, 1.99−3.49, and ≥3.5 (Vieux et al., 2019), and missing data were included as a separate category. The smoking status was categorized as “non‐smokers” (lifetime use of <100 cigarettes), “current smokers” (lifetime use of ≥100 cigarettes and who currently smoke cigarettes), and missing data as a group alone. Participants were asked if they had at least 12 alcohol drinks/1 year. Alcohol consumption was categorized as more than 12 glasses in 1 year, less than 12 glasses in 1 year, and missing data as a group alone. The height and weight of the patient were measured by trained health technicians, and the BMI of the patient was calculated by dividing the weight in kilograms by the height in meters. Diabetes, hypertension, and cancer were self‐reported via a questionnaire. Diabetes, hypertension, and cancer were defined using self‐reported diagnoses.

### 2.5 Statistical analysis

All statistical analyses were conducted using the R statistical package (R version 3.5.3) and EmpowerStats. All *p*‐values were two‐sided, and values of *p*  <  .05 were considered statistically significant. We present continuous variables as means ± standard deviations (SD) and categorical variables as totals and percentages (%). The chi‐square test was used for categorical variables. Continuous variables were first checked for normality. Whenever normal distribution criteria were met, one‐way analysis of variance (ANOVA) tests were performed, whereas when results were not normally distributed, Kruskal–Wallis tests (nonparametric ANOVA tests) were performed. Unadjusted models and multivariate adjusted models using generalized logistic regression were used to evaluate the association between HDL‐C and stroke. To explore and understand the complex relationships between HDL‐C and stroke, continuous variables were transformed into categorical variables: HDL‐C <1.04 mmol/L(40 mg/dl), 1.04–1.56 mmol/L(40–59 mg/dl), 1.56–2.07 mmol/L(60–79 mg/dl), 2.07−2.32 mmol/L (80−89 mg/dl), and ≥2.32 mmol/L (≥90 mg/dl). Furthermore, a generalized additive model was applied to find the nonlinear relationship. If a nonlinear correlation was found, we calculated the threshold effect of HDL‐C on stroke risk using a two‐piecewise linear regression model in terms of a smoothing plot. If the ratio between them appears obvious in a smooth curve, the recursive method will automatically calculate the inflection point using the maximum model likelihood. We constructed three multivariable logistic regression models: model 1, no covariates were adjusted; model 2, age, gender, and race were adjusted; and model 3, all the covariates presented in Table 1 were adjusted. Subgroup analyses stratified by gender and race were also performed.

**TABLE 1:** Baseline characteristics of participants

HDL‐C	Q1 (<1.04)	Q2 (1.04–1.56)	Q3 (1.56–2.07)	Q4 (2.07–2.32)	Q5 (≥2.32)	*p*‐Value
N	1916	4253	1853	324	297	
Age	57.11 ± 11.67	58.42 ± 11.85	59.32 ± 11.97	61.04 ± 12.06	58.16 ± 11.63	<.001
BMI	31.37 ± 6.20	29.98 ± 6.47	27.75 ± 6.52	25.83 ± 5.37	25.05 ± 5.67	<.001
Cholesterol	4.93 ± 1.23	5.08 ± 1.12	5.29 ± 0.98	5.53 ± 0.92	5.89 ± 0.97	<.001
Triglycerides	3.00 ± 2.27	1.76 ± 1.43	1.23 ± 0.64	0.98 ± 0.43	0.96 ± 0.51	<.001
HbA1c	6.25 ± 1.36	6.00 ± 1.21	5.74 ± 0.92	5.66 ± 0.91	5.49 ± 0.48	<.001
Sex (%)						<.001
Men	71.19	50.34	30.55	25.62	22.90	
Women	28.81	49.66	69.45	74.38	77.10	
Race (%)						<.001
Mexican American	19.99	18.53	12.20	11.73	6.73	
Non‐Hispanic White	44.42	40.65	41.55	42.59	49.49	
Non‐Hispanic Black	13.57	19.87	25.20	27.16	31.31	
Other race	22.03	20.95	21.05	18.52	12.46	
Level of education (%)						<.001
Less than high school	35.23	30.85	24.82	25.62	18.18	
High school	23.54	22.90	20.89	20.37	19.87	
More than high school	41.23	46.25	54.29	54.01	61.95	
Income to poverty ratio (%)						<.001
<1.99	47.91	42.49	38.26	35.19	34.68	
1.99–3.49	18.37	18.88	18.40	20.37	17.51	
>3.50	23.07	29.34	33.51	33.33	38.72	
Missing	10.65	9.29	9.82	11.11	9.09	
LDL‐C (%)						<.001
<1.5	1.72	1.50	1.19	1.54	3.70	
1.5–3.0	19.26	23.07	25.90	23.15	25.25	
3.0–4.5	14.98	21.61	22.34	20.99	19.87	
>4.5	2.14	3.69	3.24	2.78	1.68	
Missing	61.90	50.13	47.33	51.54	49.49	
Smoking status (%)						<.001
Current smokers	55.01	47.73	39.56	44.44	50.84	
Non‐smokers	44.99	52.27	60.44	55.56	49.16	
Alcohol consumption (%)						<.001
More than 12 glasses in 1 year	66.86	62.45	60.71	66.05	74.07	
Less than 12 glasses in 1 year	25.31	29.49	30.60	24.07	14.81	
Missing	7.83	8.06	8.69	9.88	11.11	
Diabetes (%)						<.001
Yes	25.37	18.39	11.23	11.11	5.72	
No	74.63	81.61	88.77	88.89	94.28	
Hypertension (%)						<.001
Yes	50.57	47.05	43.34	43.21	39.06	
No	49.43	52.95	56.66	56.79	60.94	

*Note*: Mean ± SD for continuous variables: the *p*‐value was calculated by the line regression model. (%) For categorical variables: the *p*‐value was calculated by the chi‐square test.

Abbreviations: BMI, body mass index; HbA1c, glycated haemoglobinA1c; HDL‐C, high‐density lipoprotein cholesterol; LDL‐C, low‐density lipoprotein cholesterol; Q, quintile.

## 3 RESULTS

Our study included 8643 participants after reviewing the data of 71,714 participants(women mean age: 58.35 ± 11.68 years; men mean age: 58.48 ± 12.04; 51.15% women). The flow diagram in Figure 1 shows the selection of participants. As shown in Table 1, we categorized HDL‐C levels as follows: Q1 group <1.04 mmol/L; Q2 group 1.04–1.56 mmol/L; Q3 group 1.56–2.07 mmol/L; Q4 group 2.07–2.32 mmol/L; Q5 group ≥2.32 mmol/L. There were significant differences in baseline characteristics among the HDL‐C five groups (*p*<.05). The population with higher HDL‐C levels had higher values for cholesterol, women, Whites, Blacks, education levels, alcohol consumption, and income‐to‐poverty ratio, and lower values for BMI, triglycerides, and HbA1c. Participants in the lowest group are more likely to develop diabetes and hypertension.

Table 2 shows the results of the multivariate regression analyses. In the crude model (odds ratio [OR] = 0.67, 95% confidence interval [CI]: 0.52–0.86, *p* < .05), HDL‐C was negatively correlated with stroke. After adjusting for confounding factors, this negative association was still present in the Minimally adjusted model (OR = 0.50, 95% CI: 0.38–0.66, *p* < .05) and the Fully adjusted model (OR = 0.69, 95% CI: 0.49–0.96, *p* < .05). After converting HDL‐C from a continuous variable to a categorical variable (five groups), compared with the reference group, the risk of stroke was reduced by 33%(OR = 0.67, 95% CI: 0.5094–0.8860, *p* < .05) in the Q2, by 39% (OR = 0.61, 95% CI: 0.4211–0.8962, *p* < .05) in the Q3, by 61.0% (OR = 0.39, 95% CI: 0.1906–0.8141, *p* < .05) in the Q4, and by 32% (OR = 0.68, 95% CI: 0.3388–1.3771, *p*>.05) in the highest group. Although no significant difference was seen in the highest group, the trend was significant among the five different HDL‐C groups (*p* < .05).

**TABLE 2:** The association between high‐density lipoprotein cholesterol (HDL‐C) (mmol/L) and stroke

Exposure	Crude model OR (95% CI), *p*‐value	Minimally adjusted model OR (95% CI), *p*‐value	Fully adjusted model OR (95% CI), *p*‐value
HDL‐C	0.67 (0.52, 0.86), <.05	0.50 (0.38, 0.66) <.05	0.69 (0.49, 0.96) .298
HDL‐C(quintile)			
Q1 (<1.04)	Reference	Reference	Reference
Q2 (1.04–1.56)	0.68 (0.5374, 0.8621) <.05	0.57 (0.4478, 0.7337) <.05	0.67 (0.5094, 0.8860) <.05
Q3 (1.56–2.07)	0.61 (0.4555, 0.8271) <.05	0.45 (0.3301, 0.6254) <.05	0.61 (0.4211, 0.8962) <.05
Q4 (2.07–2.32)	0.47 (0.2473, 0.9187) <.05	0.30 (0.1541, 0.5937) <.05	0.39 (0.1906, 0.8141) <.05
Q5 (≥2.32)	0.63 (0.3436, 1.1557) .14	0.46 (0.2434, 0.8555) <.05	0.68 (0.3388, 1.3771) .2866
*p* for trend	.05	0 <.05	0 <.05
Subgroup analysis stratified by sex			
Men	0.63 (0.42, 0.94) .0254	0.46 (0.30, 0.70) .0004	0.58 (0.34, 0.98) .0424
Women	0.68 (0.48, 0.95) .0263	0.53 (0.37, 0.75) .0005	0.77 (0.50, 1.18) .2316
Subgroup analysis stratified by race/ethnicity			
Mexican American	0.42 (0.17, 1.02) .0552	0.47 (0.19, 1.18) .1087	0.64 (0.21, 2.00) .4436
Non‐Hispanic White	0.45 (0.31, 0.65) <.0001	0.31 (0.20, 0.47) <.0001	0.49 (0.29, 0.82) .0070
Non‐Hispanic Black	0.92 (0.61, 1.39) .7011	0.86 (0.56, 1.33) .4993	1.03 (0.61, 1.75) .9035
Other races	0.84 (0.38, 1.85) .6575	0.67 (0.28, 1.60) .3697	0.43 (0.14, 1.35) .1463

*Note*: Fully adjusted model: All covariates are presented in Table 1. Crude model: None. Minimally adjusted model: Age, sex, race.

Abbreviations: CI, confidence interval; OR, odds ratio; Q, quintile.

**FIGURE 2:**
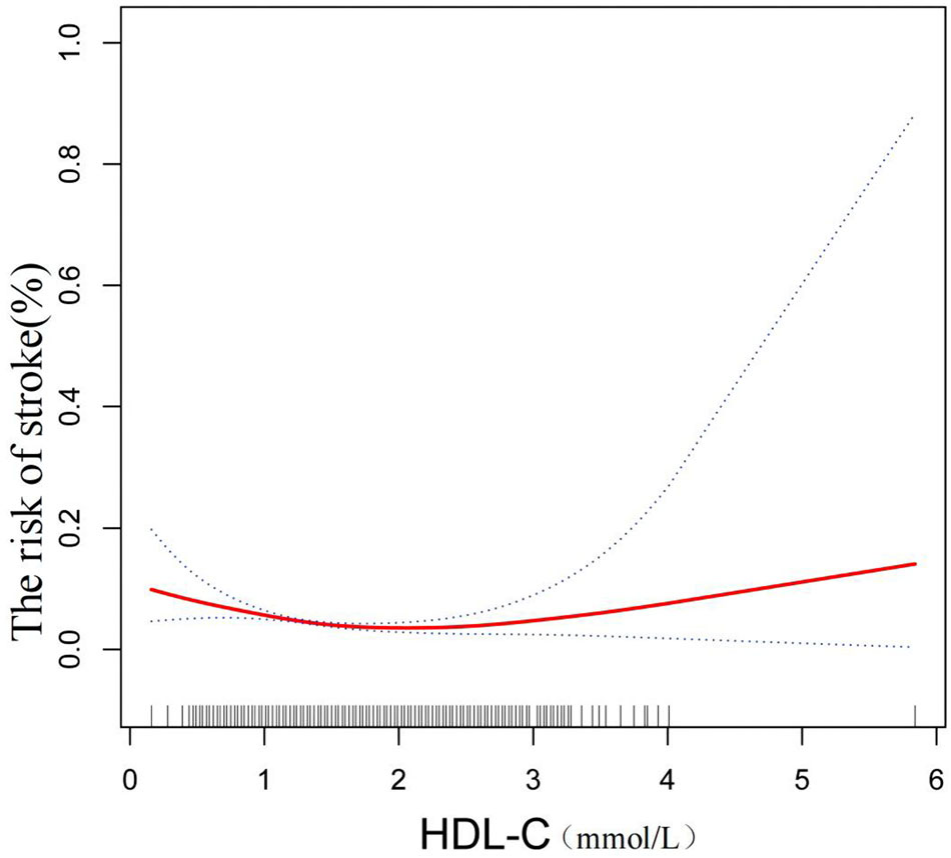
A nonlinear relationship between high‐density lipoprotein cholesterol (HDL‐C) and stroke using smooth curve fitting and generalized additive models was detected after adjusting for age, sex, race/ethnicity, low‐density lipoprotein cholesterol (LDL‐C), cholesterol, triglycerides, glycated haemoglobinA1c (HbA1c), income‐to‐poverty ratio, education level, smoking status, alcohol consumption, body mass index (BMI), diabetes, and hypertension. The inflection point of the study was 1.55 mmol/L. Below 1.55 mmol/L, the risk of stroke decreased by 64%(*p* < .05) for each unit increase of HDL‐C. Above 1.55 mmol/L, the risk of stroke increased for each unit increase, but there was no statistically significant difference(*p*>.05).The solid line and dashed line represent the estimated values and their corresponding 95% confidence intervals, respectively.

Stratified by sex and race/ethnicity in subgroup analyses(Table 2), the model 3 shows that the negative association between HDL‐C and stroke remains in men (OR = 0.58, 95% CI: 0.34–0.98, *p* < .05) and whites (OR = 0.49, 95% CI: 0.29–0.82, *p* < .05), but not in women (OR = 0.77, 95% CI: 0.50–1.18, *p*>.05), Blacks (OR = 1.03, 95% CI: 0.61–1.75, *p*>.05) and Mexican American (OR = 0.64, 95% CI: 0.21–2.00, *p*>.05). After adjusting for the above covariates, Figure 2 shows a nonlinear relationship between HDL‐C and stroke using smooth curve fitting and generalized additive models. We calculated the inflection point as 1.55 using a two‐piecewise linear regression model (Table 3). The inflection point of the study was 1.55 mmol/L. Below 1.55 mmol/L, the risk of stroke decreased by 64%(OR = 0.36, 95% CI:0.21–0.62, *p* < .05) for each unit increase of HDL‐C. Above 1.55 mmol/L, the risk of stroke increased for each unit increase, but there was no statistically significant difference(OR = 1.29, 95% CI: 0.79–2.09, *p*>.05).

**TABLE 3:** Nonlinearity addressing of high‐density lipoprotein cholesterol (HDL‐C) and stroke

Stroke	OR (95% CI), *p*‐value
HDL‐C	
Fitting model by standard logistic regression	0.69 (0.49, 0.96) .0298
Fitting model by two‐piecewise logistic regression	
Inflection point	1.55
<1.55	0.36 (0.21, 0.62) .0002
>1.55	1.29 (0.79, 2.09) .3057
*p* For log likely ratio test	.003

*Note*: Adjusted for all covariates presented in Table 1.

Abbreviations: CI, confidence interval; OR, odds ratio.

## 4 DISCUSSION

In this study, we found a negative association between HDL‐C and stroke. A nonlinear relationship between HDL‐C and stroke incidence with a point of inflection at 1.55 mmol/L was indicated. Based on subgroup analyses stratified by sex and race/ethnicity, the negative relationship of HDL‐C with stroke remained in men and Whites. This finding suggests that keeping HDL‐C levels at a slightly higher level could reduce the incidence of stroke.

Even though HDL‐C and stroke have been studied previously, the relationship between stroke and HDL‐C is limited and controversial. Three prospective studies reported an inverse association between HDL‐C levels and stroke incidence in a low level range of HDL‐C (Chei et al., 2013; Saito et al., 2017; Zhang et al., 2012). This conclusion was supported by another cohort study (Vitturi & Gagliardi, 2022). Several recent studies have shown people with extremely high HDL‐C paradoxically have high all‐cause mortality (Hirata et al., 2016, 2018; Madsen et al., 2017). Therefore, HDL‐C appears to be a double‐edged sword for atherosclerosis, possibly because both very high and low levels of HDL‐C are significantly associated with endothelial dysfunction. A prospective cohort study from China reported both low and high cumulative mean HDL‐C were associated with an increased risk of ischemic stroke and hemorrhagic stroke, a U‐shaped relationship (Li et al., 2022). Possible explanations for the U‐shaped relationship between HDL‐C and cardiovascular disease risk include genetic mutations that lead to very high HDL‐C, which contributes to adverse cardiovascular disease risk as well; extreme elevations in HDL‐C may represent dysfunctional HDL in some individuals, which in turn may increase cardiovascular risk (Singh & Rohatgi, 2018). In our research, the inverse relationship between HDL‐C and the risk of stroke at a low level range of HDL‐C was demonstrated. We did not find a positive correlation between extremely high levels of HDL‐C and stroke risk. However, extremely high levels of HDL‐C increased the height of the end curve even though the results were not statistically different. The small sample size of extremely high levels of HDL‐C could be the reason. More clinical studies are needed in the future to confirm this.

In our study, by using multiple logistic regression, stratified analysis, and trend test, HDL‐C was found to reduce the risk of stroke in both men and women. However, the benefits of HDL‐C on stroke risk attenuated after adjustment for all covariates are presented in Table 1, especially in women. Estrogen is known to contribute to cardiovascular protection by increasing HDL‐C (Tikkanen et al., 1982). HDL‐C levels are reduced to moderate levels in postmenopausal women (Matthews et al., 1989). We speculate that the larger inclusion of postmenopausal women with decreased estrogen secretion and the possible presence of residual confounders such as the current use of combined oral contraceptives, and hormone replacement therapy may be the reason. A multi‐ethnic study of people aged 45–84 in the United States showed that HDL‐C was associated with lower stroke risk; however, when interactions with race were examined, the relationship between HDL‐C and stroke was significant only in Blacks (Reina et al., 2015). We observed a stable negative correlation between HDL‐C and stroke in Whites with or without adjustment for confounders, which suggested that the HDL‐C may have an effect on stroke outcome differently in Whites than in other races. Race‐specific differences can be explained by differences in alcohol consumption, obesity, genetic factors, and other factors. Further large prospective studies are needed to elucidate the relationship between HDL‐C and stroke in the white middle‐aged and elderly people population.

The current study has several limitations. First, because of the cross‐sectional design, the causal relationship between HDL‐C and stroke was not assessed. Long‐term observational studies should be considered in future studies. Second, the study only included people over 40 years old and excluded patients with cancer, so the results cannot be used for young people and patients with cancer. Third, considering that the data sources have certain geographical and ethnic restrictions, the result of this study is only applicable to Americans. Fourth, the NHANES database does not distinguish between ischemic and hemorrhagic strokes. The relationship between stroke type and HDL‐C should be considered in future studies. Fifth, the sample size of the participants was small. In addition, our subgroup analyses and their results are exploratory since these are not established a priori. Sixth, because of the limitations of database biochemical indicators, HDL particle was not addressed in our study. Thus, further research is needed to find out the relation between HDL particles and stroke.

## 5 CONCLUSION

Our study revealed a range of negative associations between HDL‐C and stroke among people over 40 years old, especially among men and Whites. This association followed a nonlinear curve (inflection point: 1.55 mmol/L). Measurement of HDL‐C may provide a responsive biomarker for the early identification of stroke and to guide treatment.

## AUTHOR CONTRIBUTIONS

Yang Hu, Gongbo Li, and Haiyan Luo conceived and designed the study. Yang Hu, Min Song, Dongmei Wu, and Yuqing Zhang conducted the formal analysis and developed the methodology. Yang Hu and Min Song wrote the initial drafts. Dongmei Wu and Yuqing Zhang helped draft the manuscript. Gongbo Li and Haiyan Luo are the corresponding authors of this work and supervised work on the entire manuscript. All the authors read and approved the final manuscript.

## CONFLICT OF INTEREST STATEMENT

The authors have no conflicts of interest to declare.

## FUNDING INFORMATION

No funding was received for this study.

### PEER REVIEW

The peer review history for this article is available at https://publons.com/publon/10.1002/brb3.2901.

## Data Availability

The survey data are publicly available on the internet for data users and researchers throughout the world (www.cdc.gov/nchs/nhanes/).
